# Spatial light modulator aided noninvasive imaging through scattering layers

**DOI:** 10.1038/s41598-019-54048-7

**Published:** 2019-11-27

**Authors:** Saswata Mukherjee, A. Vijayakumar, Joseph Rosen

**Affiliations:** School of Electrical and Computer Engineering, Ben-Gurion University of the Negev, P.O. Box 653, Beer-Sheva, 8410501 Israel

**Keywords:** Imaging and sensing, Optical techniques

## Abstract

We propose and demonstrate a new imaging technique to noninvasively see through scattering layers with the aid of a spatial light modulator (SLM). A relay system projects the incoherent light pattern emitting from the scattering layer onto the SLM. Two coded phase masks are displayed, one after another, on the SLM to modulate the projected scattered field and the two corresponding intensity patterns are recorded by a digital camera. The above procedure helps to achieve two goals. Firstly, since the coded phase masks are digitally synthesized, the point spread function of the imaging system can be engineered such that the image retrieval becomes more reliable. Secondly, the two recorded intensity patterns are subtracted one from the other and by that the background noise of the recovered image is minimized. The above two advantages along with a modified phase retrieval algorithm enable a relatively easier and accurate convergence to the image of the covered object.

## Introduction

Imaging through scattering layers enables seeing objects present behind media such as biological tissues^1–3^, fog^4^, and other turbid media^5^. Different imaging techniques have been developed in the past to see through scattering layers which can be broadly classified into invasive and noninvasive categories depending upon whether the point spread function (PSF) of the system is measured or not. In the case of invasive techniques, it is necessary to have prior information about either the PSF of the imaging system containing the scattering layer or the phase image of the scattering layer^6–14^. Besides being invasive, these techniques are not suitable for imaging through temporally varying turbid media, like fog, or a blood flow, whose PSF varies with time. Furthermore, a high level of reconstruction noise was noticed^15,16^ in some of the recently developed 2D and 3D imaging techniques using cross-correlations between the PSF and object intensity response. Consequently, different techniques were developed to suppress the reconstruction noise by engineering the phase masks^17^, and statistical averaging^18^, which are not always practical in the case of impenetrable scattering layers. Other deconvolution techniques based on Richardson-Lucy iterative algorithm^19^ and inverse filter^20^ are invasive as well, as they require to measure the PSF in order to retrieve the image of the object.

Noninvasive imaging techniques have been developed recently to see through scatterers without knowing the PSF of the system or the phase function of the scattering layers^21–29^. In some of these noninvasive techniques, the intensity of the scattered light is autocorrelated and from this autocorrelation, the image of the concealed object is reconstructed using a phase retrieval algorithm^30,31^. While the noninvasive technique can retrieve the image without knowing the PSF, the success of the phase retrieval algorithm is highly statistical as the outcome of the phase retrieval method is dependent upon the initial guess of the iterated matrix. Consequently, the phase retrieval algorithm must run several times with different initial random patterns before the optimal result can be obtained^24^. One of the main requirements for the phase retrieval based noninvasive imaging techniques to be successful is that the phase variation of the scattering layers must be chaotic such that the autocorrelation of the system PSF yields a sharply peaked function with a constant distribution as a background. This requirement highly limits the applicability of the technique. In a formal notation, for incoherent illumination and for object intensity *O*, the intensity distribution recorded by the camera is *O* * *I*_*PSF*_, where *I*_*PSF*_ is the system PSF and ‘*’ is the sign of 2D convolution. In^23,24^, it is suggested to compute the autocorrelation (*O* * *I*_*PSF*_) ⊗ (*O* * *I*_*PSF*_), where ⊗ represents the 2D correlation operator. The same autocorrelation can be written as (*O* ⊗ *O*) * (*I*_*PSF*_ ⊗ *I*_*PSF*_)^32^, Under the assumption that (*I*_*PSF*_ ⊗ *I*_*PSF*_), is equal to a delta function plus a constant (*δ* + Constant), the autocorrelation of the camera output is approximately (*O* ⊗ *O* + Constant) and hence the object distribution can be retrieved by a constant removal procedure and a phase retrieval algorithm. However, the assumption that (*I*_*PSF*_ ⊗ *I*_*PSF*_) ≈ *δ* + Constant is sometimes problematic. First, the autocorrelation of a positive chaotic PSF has positive non-constant background distribution with double the size of *I*_*PSF*_. Second, the width of the delta-like function in certain setups is dependent on the properties of the scattering layer and hence the approximation to a narrow delta-like function is limited only to certain scatterers. Because of these two effects, the approximation (*I*_*PSF*_ ⊗ *I*_*PSF*_) ≈ *δ* + Constant is sometimes not established well, and hence the input of the phase retrieval algorithm is far from the ideal (*O* ⊗ *O*) Consequently, the output of the algorithm might not converge to the required image of *O*.

In this study, we present a new approach to extend the applicability of the phase retrieval based noninvasive imaging techniques for imaging through various types of scattering layers. The main concept of the proposed method is to project the scattered light emitted from the scattering layer on a computer-controlled coded phase mask (CPM) which is used as a second scattering mask. By doing that, we intend to achieve two goals. First, since the second mask is synthesized digitally, we have better control on the properties of the combined scattering medium, such that a better approximation to the delta function can be obtained from the autocorrelation of the PSF. Second, in our proposed procedure, two camera shots, with two different CPMs, are captured and subtracted one from the other. Hence, the obtained superposed distribution *O* * [*I*_*PSF*1_ *− I*_*PSF*2_] is bi-polar with negligible bias term. Consequently, the background of the autocorrelation of the PSF is much lower than the background obtained from the autocorrelation of a positive function regardless whether this later background is constant or not. These two improvements in the original method bring the autocorrelation of the system response closer to the autocorrelation of the hidden object. Thus, the proposed method increases the chances of the phase retrieval algorithm to converge to an image, which is more similar to the original object.

## Methods

**Figure 1:**
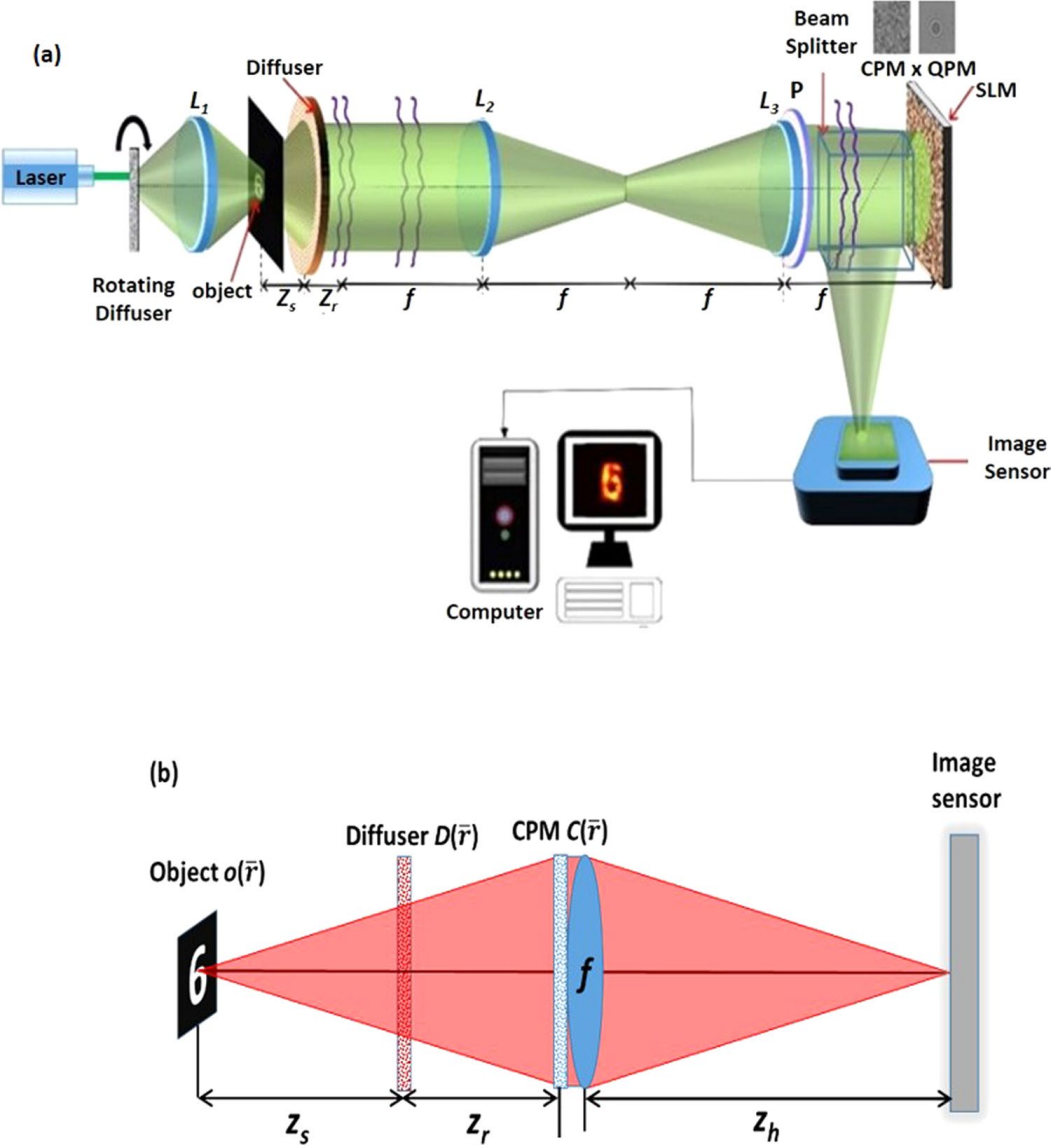
(**a**) Optical configuration of the imaging setup. (**b**) Simplified optical scheme of (**a**) used for the analysis of the setup; CPM – Coded phase mask; *L*_1_, *L*_2_, *L*_3_ – Refractive lenses; P – Polarizer; SLM – Spatial light modulator and QPM – Quadratic phase mask.

The optical configuration of the proposed setup is shown in Fig. 1(a). A spatially incoherent light^33^ critically illuminates an object using a lens *L*_*1*_. The light diffracted from the object is incident on a diffuser used as the scattering layer and located at a distance of *z*_*s*_ from the object. A relay system projects the light pattern obtained at a distance of *z*_*r*_ from the diffuser onto a phase-only spatial light modulator (SLM). The relay system can either be constructed from two identical lenses *L*_*2*_ and *L*_3_ with an overall magnification of 1, like in our experiment or if required, from different lenses with a magnification different from 1. The phase mask displayed on the SLM is a modulo-2π phase addition of the CPM, synthesized using modified Gerchberg-Saxton algorithm (GSA)^34^, and a quadratic phase mask (QPM) with the focal distance *f* used as a diffractive lens for focusing the light on an image sensor. The light modulated by the SLM is incident on the image sensor located at a distance of *z*_*h*_ from the SLM.

### System analysis

The goal of the following mathematical formalism is to obtain the expression of the intensity distribution over the sensor plane for any arbitrary object and diffuser functions. Knowing this distribution might improve our understanding of the parameters which contribute to the quality of the reconstructed images. A simplified scheme for calculating the intensity over the sensor plane is given in Fig. 1(b), in which the QPM is symbolized by a lens with a focal length of *f*. Furthermore, the illuminating sub-system and the relay are omitted since these components have no influence on the mathematical analysis. We choose to represent the object intensity distribution $o(\bar{r})$ as a series of delta functions, whereas the diffuser $D(\bar{r})$ and the CPM $C(\bar{r})$ are represented as Fourier series of linear phases, as following,

1 $$o(\bar{r})=\mathop{\sum }\limits_{j=1}^{N}{a}_{j}\delta (\bar{r}-{\bar{r}}_{j}),D(\bar{r})=\mathop{\sum }\limits_{j=-\infty }^{\infty }{b}_{j}\,\exp (i2\pi {\bar{\nu }}_{j}\cdot \bar{r}),C(\bar{r})=\mathop{\sum }\limits_{j=-\infty }^{\infty }{c}_{j}\,\exp (i2\pi {\bar{\nu }}_{j}\cdot \bar{r})\cdot $$

Assume the imaging Eq. (1/u) + (1/*z*_*h*_) = 1/*f* is fulfilled between the object and the sensor planes, where *u* = *z*_*s*_ + *z*_*r*_ and the distances *z*_*s*_, *z*_*r*_ and *z*_*h*_ are defined in Fig. 1(b). For a single point source at ${\bar{r}}_{j}$ and a single linear phase at each of the two phase masks $D(\bar{r})$ and $C(\bar{r})$, the image on the sensor is the image of the source point but shifted according to the parameters of the linear phases. This shift can be expressed by the convolution operator as the following,

2 $$I({\bar{r}}_{o})={a}_{j}\delta ({\bar{r}}_{o}-\frac{{z}_{h}{\bar{r}}_{j}}{u})\,\ast \,{|{b}_{k}\delta ({\bar{r}}_{o}-\frac{\lambda {z}_{h}{z}_{s}{\bar{\nu }}_{k}}{u})\ast {c}_{l}\delta ({\bar{r}}_{o}-\lambda {z}_{h}{\bar{\nu }}_{l})|}^{2},$$

where λ is the central wavelength of the illumination light. Equation (2) is obtained under the assumption of an incoherent optical system with infinite aperture size^35^. The various shifts of the second and third Delta functions are obtained from ray analysis^35^ through thin prisms which their transparent function is a linear phase. For the entire points of the object and for the entire linear phases composing the phase masks, the intensity on the camera is a convolution between the three series, as follows,

3 $$I({\bar{r}}_{o})=\mathop{\sum }\limits_{j}^{N}{a}_{j}\delta ({\bar{r}}_{o}-\frac{{z}_{h}{\bar{r}}_{j}}{u})\,\ast \,{|\mathop{\sum }\limits_{j}^{\infty }{b}_{j}\delta ({\bar{r}}_{o}-\frac{\lambda {z}_{h}{z}_{s}{\bar{\nu }}_{j}}{u})\ast \mathop{\sum }\limits_{j}^{\infty }{c}_{j}\delta ({\bar{r}}_{o}-\lambda {z}_{h}{\bar{\nu }}_{j})|}^{2}\cdot $$

The most left series represent the object according to Eq. (1), whereas the next two series represent the Fourier transforms of the diffuser with the scaling operator of *ν*[*u/λz*_*s*_*z*_*h*_], and of the CPM with the scaling operator of *ν*[1/*λz*_*h*_]. The scaling operator is defined by the equation *ν*[*α*]*f*(*x*) = *f*(*αx*). Therefore, the intensity on the camera is,

4 $$\begin{array}{ccc}I({\bar{r}}_{o}) & = & o({\bar{r}}_{o})\,\ast \,{|\nu [\frac{u}{\lambda {z}_{h}{z}_{s}}]{\mathfrak{F}}\{D(\bar{r})\}\ast \nu [\frac{1}{\lambda {z}_{h}}]{\mathfrak{F}}\{C(\bar{r})\}|}^{2}\\  & = & {A}_{o}o({\bar{r}}_{o})\,\ast \,{|{\mathfrak{F}}\{D(\frac{\lambda {z}_{h}{z}_{s}\bar{r}}{u})\cdot C(\lambda {z}_{h}\bar{r})\}|}^{2},\end{array}$$

Where ${\mathfrak{F}}$ the 2D Fourier transform operator and *A*_*o*_ is a constant. Based on Eq. (4), the intensity response of the system to a point source in the origin known as the PSF is,

5 $${I}_{PSF}({\bar{r}}_{o})={|{\mathfrak{F}}\{D(\frac{\lambda {z}_{h}{z}_{s}\bar{r}}{u})\cdot C(\lambda {z}_{h}\bar{r})\}|}^{2}\cdot $$

It should be noted that Eq. (4) can be generalized to *K* independent thin scattering layers positioned in the space between the hidden object and the lens in Fig. 1(b), where the CPM is one of them. In other words, for a series of *K* thin diffusers, in which each diffuser Γ_*k*_ is located at a distance *z*_*s,k*_ from the object, the intensity on the camera is,

6 $$I({\bar{r}}_{o})={A}_{o}o({\bar{r}}_{o})\ast {|{\mathfrak{F}}\{\mathop{\prod }\limits_{k=1}^{K}{\Gamma }_{k}(\frac{\lambda {z}_{h}{z}_{s,k}\bar{r}}{u})\}|}^{2},$$

Equation (6) implies that the present analysis can be applied for imaging through series of *K* thin layers^36,37^ assuming that the contribution of the entire reflections from these layers can be neglected.

### Engineering of the autocorrelation profile using CPM

Equation (5) indicates that the intensity response obtained by the given diffuser can be modified by introducing a CPM into the system. As explained in the introduction, the reconstruction algorithm is efficient if the autocorrelation of *I*_*PSF*_ is as close as possible to a delta function. Hence, the CPM is introduced into the system in order to bring the autocorrelation of *I*_*PSF*_ as close as possible to a delta function. Recall that the autocorrelation of a positive random function includes a non-constant background distribution around the delta-like response, we suggest acquiring two camera shots with two independent CPMs and to subtract one response from the other. By doing that, one can get a bipolar PSF with a negligible bias, in which its autocorrelation is lack of the undesired background distribution. Therefore, two intensity patterns are recorded, and one is subtracted from the other to obtain a bipolar intensity pattern *H*_*OBJ*_ given by,

7 $$\begin{array}{rcl}{H}_{OBJ}({\bar{r}}_{0}) & = & {I}_{OBJ,1}({\bar{r}}_{0})-{I}_{OBJ,2}({\bar{r}}_{0})\\  & = & o({\bar{r}}_{o})\ast [{|{\mathfrak{F}}\{D(\frac{\lambda {z}_{h}{z}_{s}\bar{r}}{u})\cdot {C}_{1}(\lambda {z}_{h}\bar{r})\}|}^{2}-{|{\mathfrak{F}}\{D(\frac{\lambda {z}_{h}{z}_{s}\bar{r}}{u})\cdot {C}_{2}(\lambda {z}_{h}\bar{r})\}|}^{2}]\end{array}$$

where ${C}_{i}(\bar{r})$ is the phase function of the *i*^*th*^ CPM.

**Figure 2:**
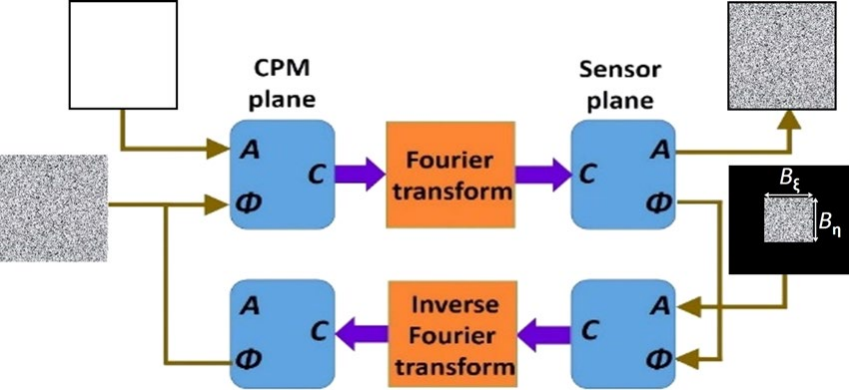
Modified Gerchberg Saxton algorithm for synthesizing coded phase masks with different scattering degrees.

**Figure 3:**
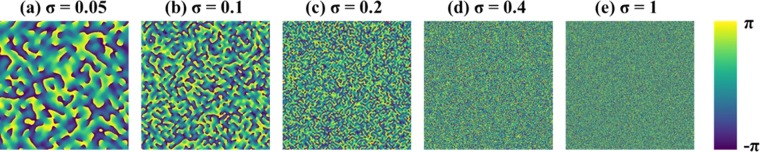
Phase images of the pseudorandom coded phase masks with different scattering degrees synthesized by GSA.

The GSA algorithm shown schematically in Fig. 2 is used to synthesize the CPMs with different degrees of scattering. The GSA is implemented between the planes of the CPM and the sensor to obtain on the sensor plane an intensity pattern with a limited area. Since the sensor plane is the spectral domain of the CPM, the area limit imposed on the sensor plane controls the maximal scattering angle of the synthesized CPM. Assuming the width *B*_ξ_ along ξ is equal to the height *B*_η_ along η, the degree of scattering *σ* is defined as the ratio *B/B*_*max*_, where *B* = *B*_ξ_ = *B*_η_ and *B*_*max*_ is the maximum possible value of *B*. The phase images of the CPMs synthesized using GSA for *σ* = 0.05, 0.1, 0.2, 0.4 and 1 are shown in Fig. 3(a–e), respectively.

Apparently, the basic assumption of the phase-retrieval-like algorithms in which the autocorrelation of the PSF has a shape of a delta function is not always satisfied. We show the difficulties with this assumption by computing the PSF for the above mentioned five values of *σ*, and for two possible setups, with and without the diffractive converging lens with the focal length *f* [the QPM in Fig. 1(a) and the lens in Fig. 1(b)]. The purpose of the QPM is to focus the modulated light onto the sensor and by that to increase the SNR of the imaging. Moreover, as shown in the following, the QPM guarantees an autocorrelation of the PSF with the same peak width, regardless the value of *σ*. The case without the QPM is considered herein because this is the configuration of several other experiments^23–27^. If the basic assumption of the sharply peaked autocorrelation of the PSF is not valid, the convergence of the phase retrieval algorithm to the real hidden object becomes rare and when it happens an automatic algorithm cannot know if it is the true expected reconstruction. Next, we show by computer simulation and later by laboratory experiments that by introducing CPM into the system, the autocorrelation of the new PSF becomes more similar to the desired delta-like function.

**Figure 4:**
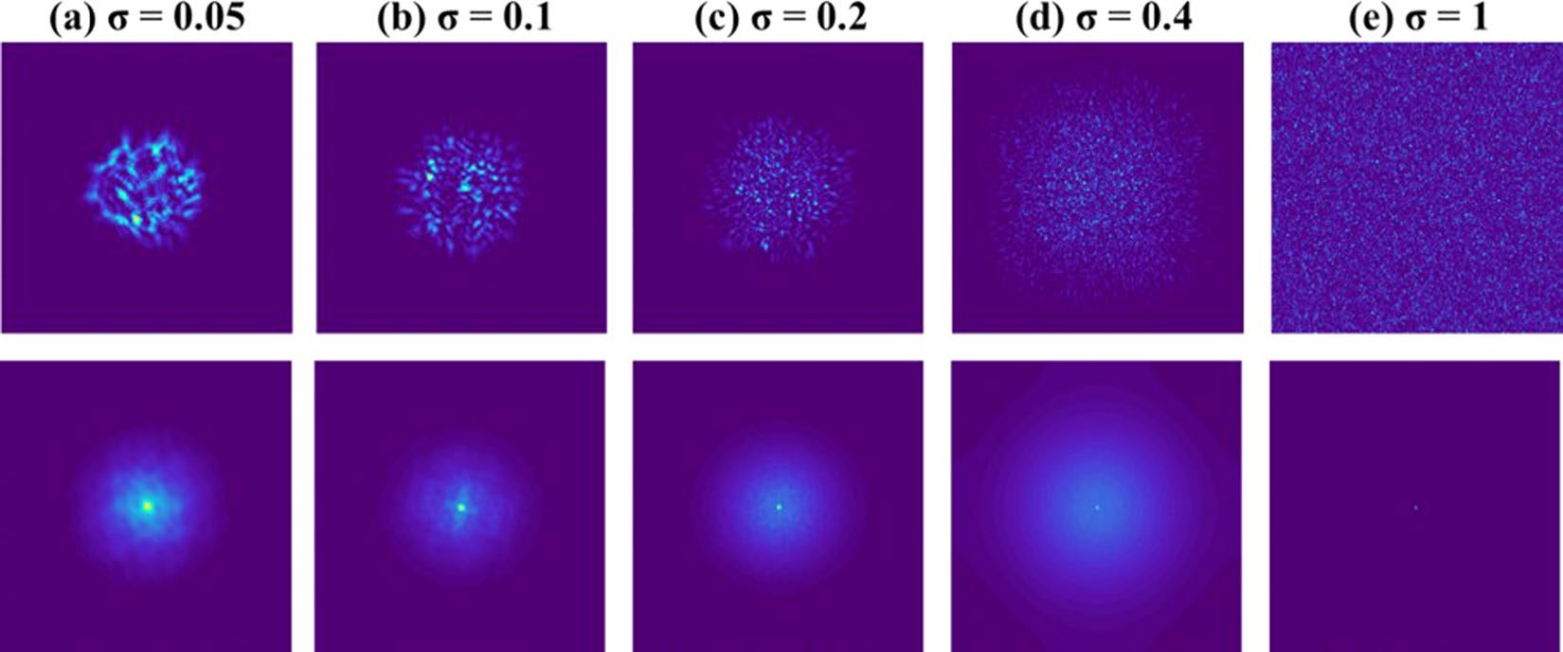
Images of simulated PSFs (top row) and their respective autocorrelation images (bottom row) when only a single scattering medium is positioned in the lensless system without the QPM. Every image consists of 500 × 500 pixels.

**Figure 5:**
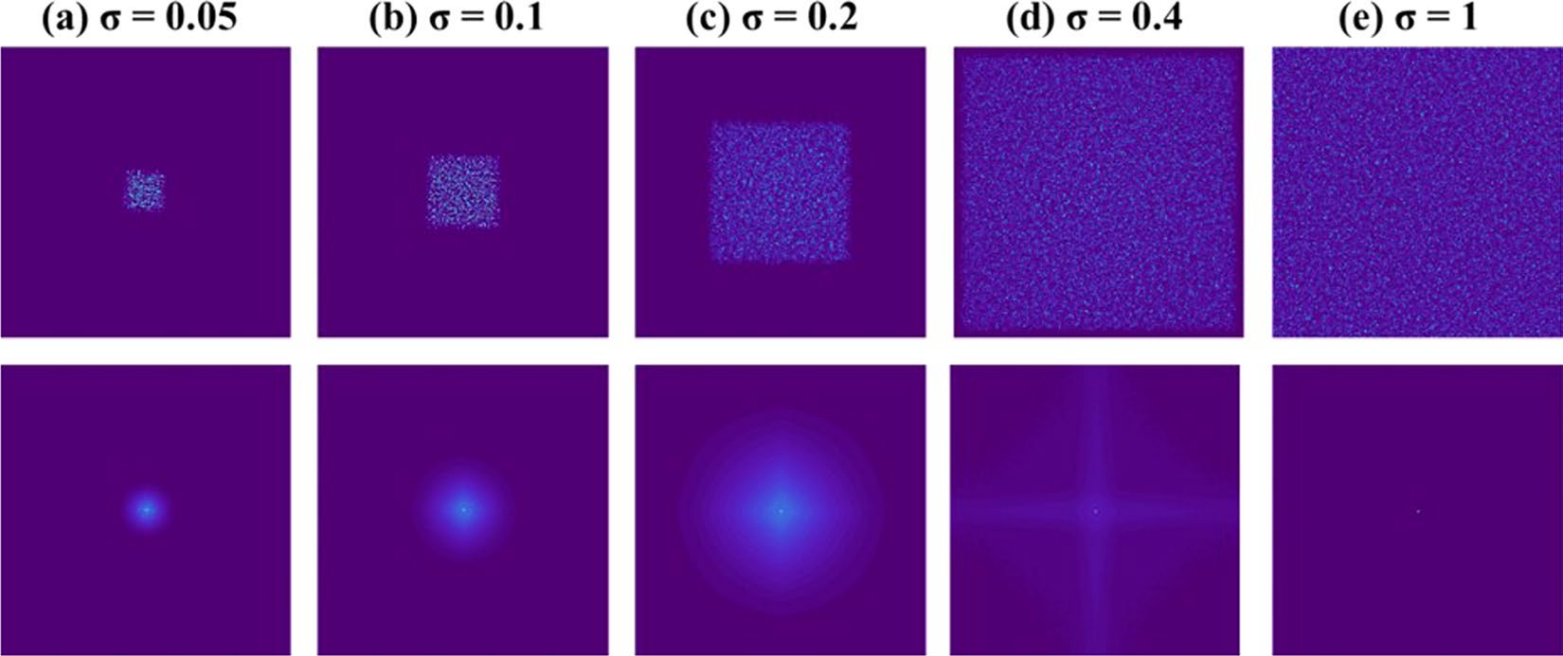
Images of simulated PSFs (top row) and their respective autocorrelation images (bottom row) when a single scattering medium with attached QPM is positioned in the system. Every image consists of 500 × 500 pixels.

**Figure 6:**
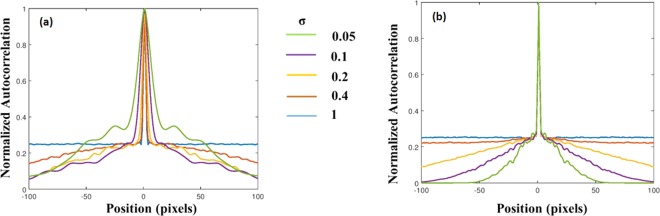
Plot of the normalized cross-sections of (*I*_*PSF*_ ⊗ *I*_*PSF*_) for different values of σ for (**a**) a lensless single scattering medium of Fig. 4 and (**b**) a single scattering medium with QPM of Fig. 5.

A point object is introduced into the simulated system, and its complex amplitude propagates through the different optical components of the optical configuration shown in Fig. 1(b), excluding the Diffuser $D(\bar{r})$. The distances used in the simulation are *z*_*s*_ = 0 cm, *z*_*r*_ = 16 cm, *z*_*h*_ = 12 cm and *f* = 6.9 cm. For the lensless case^21–26^, the distances are *z*_*r*_ = 5 cm and *z*_*h*_ = 5 cm. In both cases, with and without the QPM, the only single scattering medium is positioned at the SLM plane and this medium is tested for 5 different values of *σ*. For both configurations, the intensity patterns (*I*_*PSF*_) and their corresponding autocorrelation profiles (*I*_*PSF*_ ⊗ *I*_*PSF*_) are depicted for different *σ*, as shown in Fig. 4 and Fig. 5. The plots of the cross-section of (*I*_*PSF*_ ⊗ *I*_*PSF*_) for different values of *σ* for the above two cases are shown in Fig. 6(a,b), respectively. From these results, it is clear that the auto-correlation profile strongly depends on the scattering degree, and in each profile the sharply peaked function is accompanied with some background distribution, irrespective to the QPM presence.

**Figure 7:**
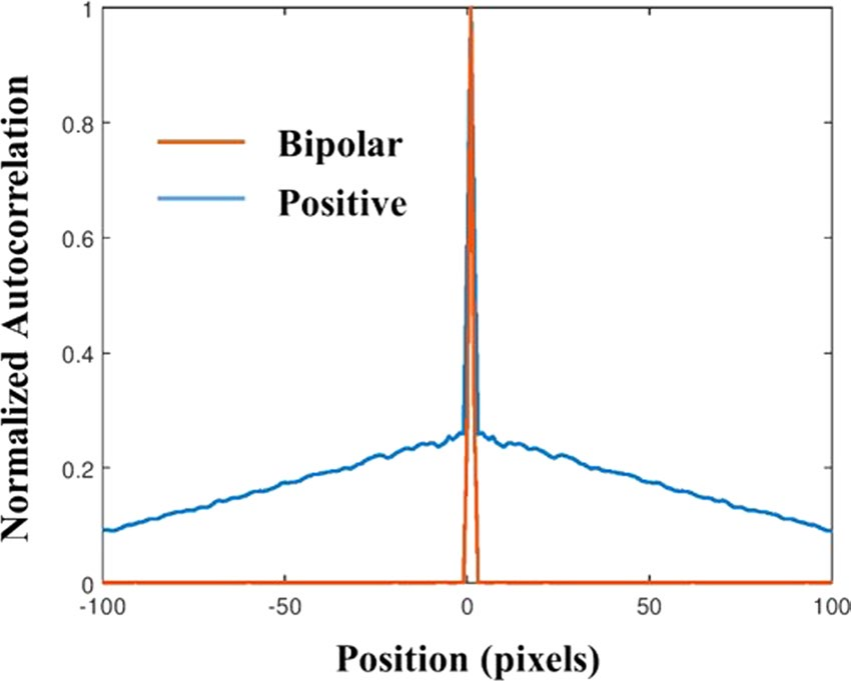
Autocorrelation profile of positive PSF (blue) and bipolar PSF (orange) for σ = 0.2 when QPM is attached to the diffuser.

From Fig. 6(a), it is evident that without QPM, as much as the scattering degree has increased the autocorrelation is narrower. This dependency is clear since the PSF in the lensless case is a free-space diffraction in the Fresnel regime. That means the PSF is approximately a square magnitude of the convolution between the function of scattering layer and the quadratic phase function of the free space^32^. Such convolution does not yield a PSF with a different bandwidth than that of the scattering layer. Hence, the smallest feature size of the PSF, and the width of the autocorrelation peak in the lensless case are equal to the feature size of the diffuser and inversely proportional to σ. The conclusion from this observation is that in the lensless case^23,24^ the assumption (*I*_*PSF*_ ⊗ *I*_*PSF*_) ≈ *δ* + Constant is valid for relatively strong scatterers only. On the other hand, Fig. 6(b) indicates that the width of the autocorrelation peak is independent of the scattering degree. In other words, because of the Fourier relation between the scattering media and the camera plane, the intensity pattern *I*_*PSF*_ obtained on the camera plane is a speckle pattern in which the feature size of the speckles is dependent on the sizes *λ, z*_*h*_*, D* but independent on σ. Since the feature size of the speckles dictates the width of the autocorrelation peak, this width is independent of σ. Equation (4) indicates that introducing the CPM into the system does not change the independency of the autocorrelation peak in σ. In both cases of Fig. 6(a,b), the central peak is accompanied by a background, which becomes wider by increasing the scattering degree. Therefore, to guarantee an appropriate convergence of the phase retrieval algorithm, one should eliminate the background. Following previous methods of eliminating the background^17^, we display one by one on the SLM two independent CPMs and subtract one intensity response from the other. The result is a bipolar PSF that its autocorrelation is close to a delta function with a negligible background, as is shown in Fig. 7. Note that this background removing process is not limited to a constant background^24^ and works well for any type or any width of background as long as the statistical properties of the PSF are the same for both camera shots.

### Phase retrieval algorithm

Assuming the autocorrelation of the PSF is sharply peaked function, the autocorrelation of the object intensity pattern on the image sensor in the presence of the scattering layers, with or without QPM, can be expressed as,

8 $$\begin{array}{rcl}{I}_{OBJ}\otimes {I}_{OBJ} & = & ({I}_{PSF}\ast o(\bar{r}))\otimes ({I}_{PSF}\ast o(\bar{r}))\\  & = & ({I}_{PSF}\otimes {I}_{PSF})\ast (o(\bar{r})\otimes o(\bar{r}))\\  & \cong  & (o(\bar{r})\otimes o(\bar{r}))+B(\bar{r}),\end{array}$$

where $B(\bar{r})$ is a background function. In the proposed technique, the QPM can be used to engineer the autocorrelation profile of the PSF to be as sharp as possible as is implied from the differences between Fig. 6(a,b). The CPM can be used to engineer the width of the background, which can be removed almost completely, regardless of its width, by the use of the bipolar intensity pattern as is shown in Fig. 7. Based on Eq. (7) the bipolar PSF is,

9 $$\begin{array}{rcl}{I}_{PSF}({\bar{r}}_{0}) & = & {I}_{PSF,1}({\bar{r}}_{0})-{I}_{PSF,2}({\bar{r}}_{0})\\  & = & {|{\mathfrak{F}}\{D(\frac{\lambda {z}_{h}{z}_{s}\bar{r}}{u})\cdot {C}_{1}(\lambda {z}_{h}\bar{r})\}|}^{2}-{|{\mathfrak{F}}\{D(\frac{\lambda {z}_{h}{z}_{s}\bar{r}}{u})\cdot {C}_{2}(\lambda {z}_{h}\bar{r})\}|}^{2}\end{array}$$

Thus, the autocorrelation of the scattered light can be represented as:

10 $$\begin{array}{c}{H}_{OBJ}\otimes {H}_{OBJ}=({I}_{OBJ,1}-{I}_{OBJ,2})\otimes ({I}_{OBJ,1}-{I}_{OBJ,2})\\ ={I}_{OBJ,1}\otimes {I}_{OBJ,1}-{I}_{OBJ,1}\otimes {I}_{OBJ,2}-{I}_{OBJ,2}\otimes {I}_{OBJ,1}+{I}_{OBJ,2}\otimes {I}_{OBJ,2}\\ =[o(\bar{r})\,\ast \,{I}_{PSF,1}]\otimes [o(\bar{r})\,\ast \,{I}_{PSF,1}]-2B(\bar{r})+[o(\bar{r})\,\ast \,{I}_{PSF,2}]\otimes [o(\bar{r})\,\ast \,{I}_{PSF,2}]\\ =[o(\bar{r})\otimes o(\bar{r})]\ast [{I}_{PSF,1}\otimes {I}_{PSF,1}]-2B(\bar{r})+[o(\bar{r})\otimes o(\bar{r})]\ast [{I}_{PSF,2}\otimes {I}_{PSF,2}]\\ \cong {a}_{1}[o(\bar{r})\otimes o(\bar{r})]\ast \delta (\bar{r})+B(\bar{r})-2B(\bar{r})+{a}_{2}[o(\bar{r})\otimes o(\bar{r})]\ast \delta (\bar{r})+B(\bar{r})\\ \cong a[o(\bar{r})\otimes o(\bar{r})]\cong a{{\mathfrak{F}}}^{-1}\{{|{\mathfrak{F}}\{o(\bar{r})\}|}^{2}\}\end{array}$$

where ${\mathfrak{F}}$^−1^ indicates the inverse 2D Fourier transform and *a*_1_, *a*_2_, *a* are constants. In Eq. (10), it is assumed that regardless of the precise CPM distribution, as long as the statistical parameters are the same for the two CPMS, the background function $B(\bar{r})$ is approximately the same for the two autocorrelations of the object intensities and for the cross-correlation between them. Based on Eq. (10) the magnitude of the object’s spectrum is approximately,

11 $$|{\mathfrak{F}}\{o(\bar{r})\}|\cong {a}^{-1}\sqrt{{\mathfrak{F}}\{{H}_{OBJ}\otimes {H}_{OBJ}\}}$$

The spectral magnitude of the object is fed into the phase retrieval algorithm^30^, where the missing spectral phase is evaluated by this iterative algorithm. The size of the object is approximately half of the size of the autocorrelation pattern given in Eq. (10) divided by the system magnification *M*_*T*_. Hence, by measuring the size of the autocorrelation pattern 2*M*_*T*_ × (*w*_*x*_,*w*_*y*_), one can estimate the limiting window of the size (*w*_*x*_,*w*_*y*_) on the object plane of the iterative algorithm.

**Figure 8:**
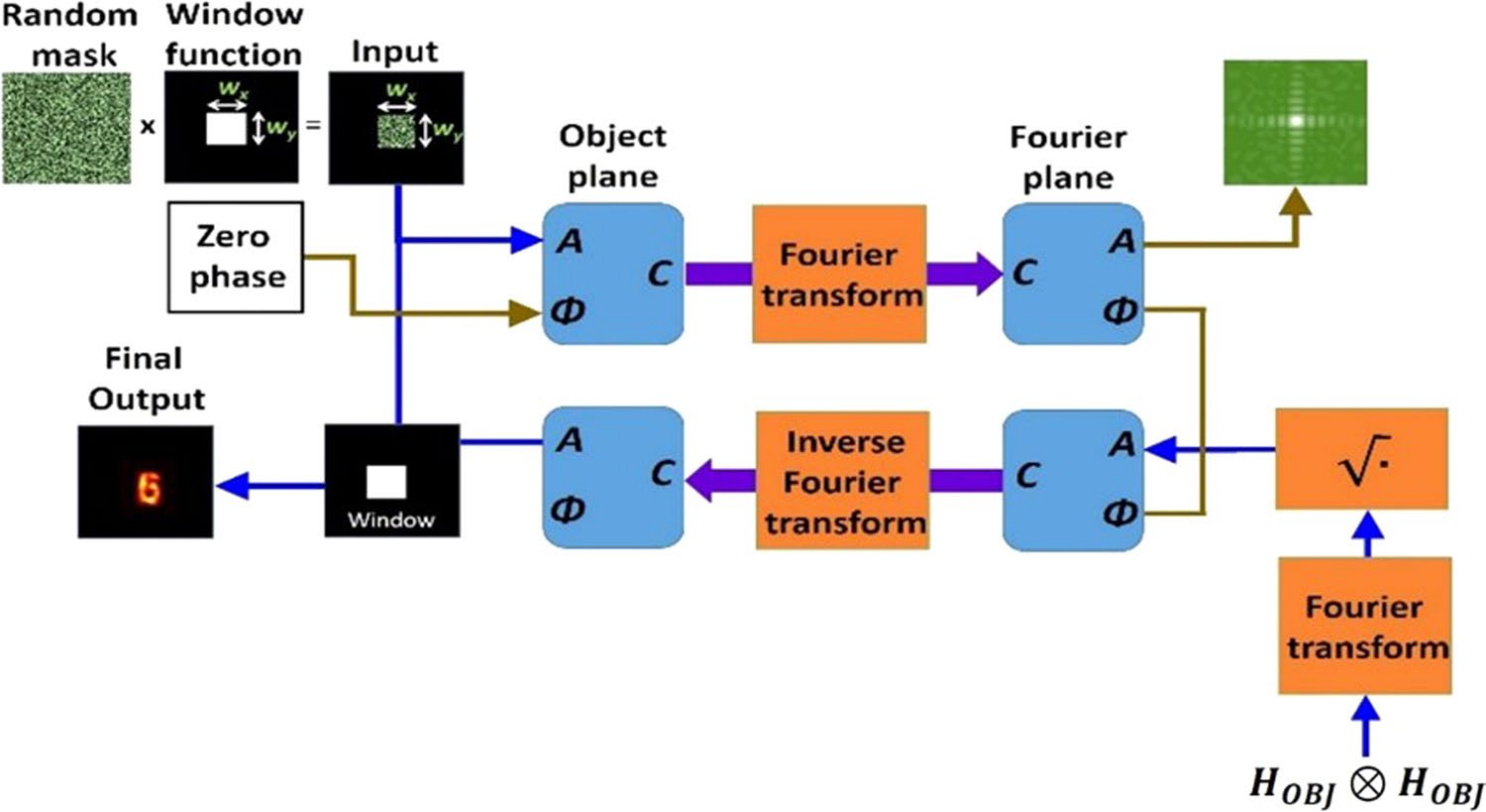
Modified phase retrieval algorithm for retrieving the image of the hidden object.

The phase retrieval algorithm shown in Fig. 8 begins with an initial random magnitude matrix over the limited window and zero values over the rest of the object plane. The object plane information is Fourier transformed and the magnitude is replaced by $\sqrt{{\mathfrak{F}}\{{H}_{OBJ}\otimes {H}_{OBJ}\}},$ while the phase information is retained. The modified complex amplitude is inverse Fourier transformed and non-negative and real values constraints are applied in addition to the window constraint mentioned above. This process is repeated until hopefully an image similar to the object is retrieved.

## Results

The experimental setup for the proposed method is shown in Fig. 1(a). Light emitted from a solid-state laser (*λ* = 532 nm) is passed through a rotating diffuser. The rotating diffuser was constructed using a thin polycarbonate sheet rotated by a DC motor to minimize the spatial coherence of the incoming light. From the diffuser, it was collected by a refractive lens *L*_1_ to critically illuminate the object. Initially, we had used a pinhole with a diameter of 100μm in the object plane to validate our assumptions and later, we used a more complex object. The light diffracted by the object passed through a constant ground glass diffuser (Thorlabs DG20-1500-MD, SM2-Mounted N-BK7 Ground Glass Diffuser) with 1500 grit and a thickness of 2 mm, located at a distance of about 5 mm from the object. The diffused light was projected on a phase-only reflective SLM [Jasper EDucation Kit (EDK) – JD955B, 1920 × 1080 pixels, 6.54 *μm* pixel pitch] by a relay system comprising of two identical refractive lenses *L*_2_ and *L*_3_ with a diameter of 5 *cm* and focal length of *f* = 10 *cm*. The light emitted from the diffuser further propagated a distance of *z*_*r*_ = 16 *cm* before reaching the relay system, which projected the light onto the SLM plane. A polarizer P placed beyond *L*_3_ was used to polarize the light along the orientation of the SLM active axis to maximize the modulation efficiency. A quadratic phase mask with a focal length of 6.9 cm was multiplied with CPM synthesized using GSA and both were displayed on the SLM. The reflected light from the SLM was recorded by an image sensor (GigE vision GT Prosilica, 2750 × 2200 pixels, 4.54 *μm* pixel pitch) located at a distance of *z*_*h*_ = 11.7 *cm*, from the SLM.

**Figure 9:**
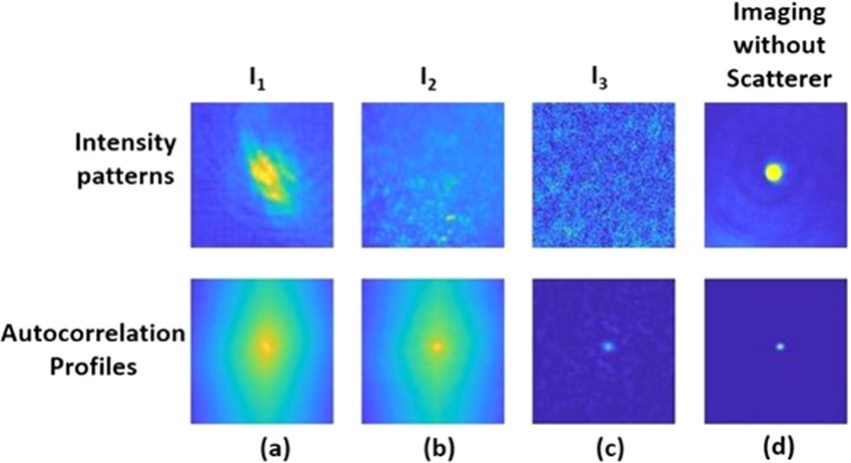
Images of intensity patterns and their respective autocorrelations, (**a**) *I*_1_ denotes intensity pattern recorded by the sensor when only QPM is displayed on the SLM, (**b**) *I*_2_ denotes intensity pattern recorded by the sensor when QPM and CPM are displayed on the SLM, (**c**) *I*_3_ denotes a bipolar pattern obtained by *I*_2_−*I*_2*b*_ and (**d**) direct imaging of the pinhole without any physical diffuser. Every image consists of 500 × 500 pixels.

In the first experiment with the pinhole of 100 *µm* diameter, three cases were studied. In case 1, only a quadratic phase mask was displayed on the SLM and the intensity pattern (*I*_1_) at the sensor plane was recorded. In case 2, CPM with a scattering degree of σ = 0.2 was displayed with the QPM and the intensity pattern (*I*_2_) was recorded. In case 3, a second independent CPM was combined with the QPM and another intensity pattern (*I*_*2b*_) was recorded. A bipolar pattern (*I*_3_ = *I*_2_ − *I*_2*b*_) was calculated and stored. The patterns and their autocorrelation for the three cases are shown in Fig. 9. It is clearly seen that the autocorrelation result of the bipolar pattern in Fig. 9(c) has the closest resemblance to the autocorrelation of the pinhole image shown in Fig. 9(d).

**Figure 10:**
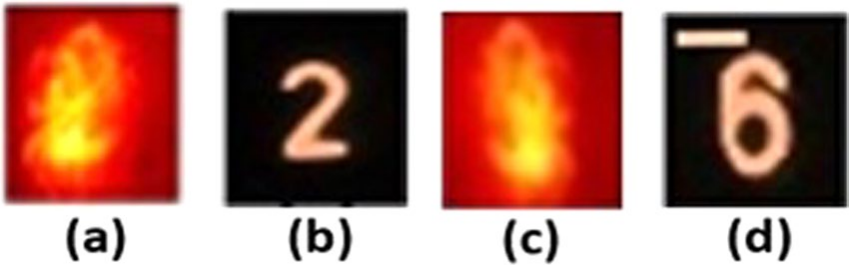
(**a–c**) Intensity pattern on the sensor plane with the diffuser and only QPM on the SLM for the digit ‘2’ and for the digit ‘6’, respectively, (**b–d**) direct images of the object without the diffuser and with QPM displayed on the SLM. Scale bar: 350 *μm*.

In the next experiment, the digits ‘2’ and ‘6’ from group 4 of United States Air Force (USAF) resolution target were tested. The objects were placed one by one at the object plane behind the static diffuser. First, only QPM was displayed on the SLM and the intensity patterns shown in Fig. 10(a,c) were recorded by the sensor. In comparison to Fig. 10(b,d) taken without the diffusers, the digits in Fig. 10(a,c) are not recognizable and hence a method of image recovery is required.

**Figure 11:**
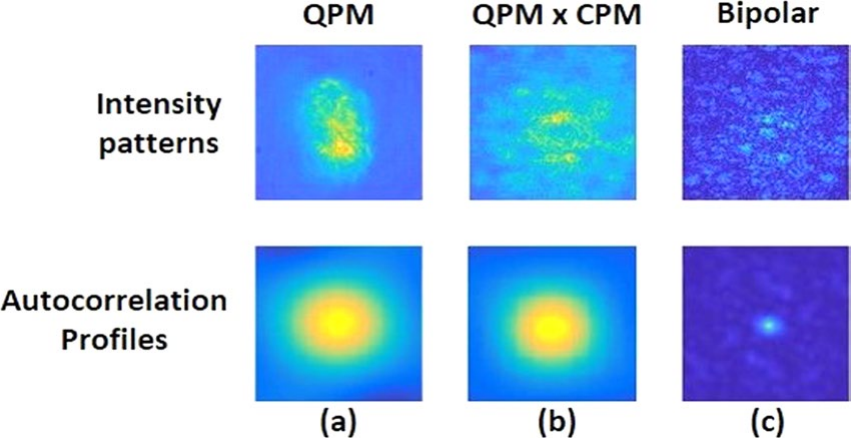
Images of intensity patterns correspond to digit 6 from group 4 of United States Air Force (USAF) resolution target, and their respective autocorrelation profiles. (**a**) Intensity pattern recorded by the sensor and its autocorrelation pattern when QPM is displayed on the SLM. (**b**) Intensity pattern recorded by the sensor and its autocorrelation pattern when QPM and CPM are displayed on the SLM. (**c**) Bipolar pattern and its autocorrelation pattern. Every image consists of 500 × 500 pixels.

Next, two intensity patterns corresponding to two different CPMs both with σ = 0.2 were recorded, where the hidden object is the digit ‘6’. One pattern was subtracted from the other to yield the bipolar pattern. The bipolar pattern indeed reduces the bias term from the autocorrelation as is shown in Fig. 11(c) in comparison to the other two cases of Fig. 11(a) without CPM and of Fig. 11(b) with a single CPM. Thus, a significant improvement of the reconstruction results over the other two cases is expected.

**Figure 12:**
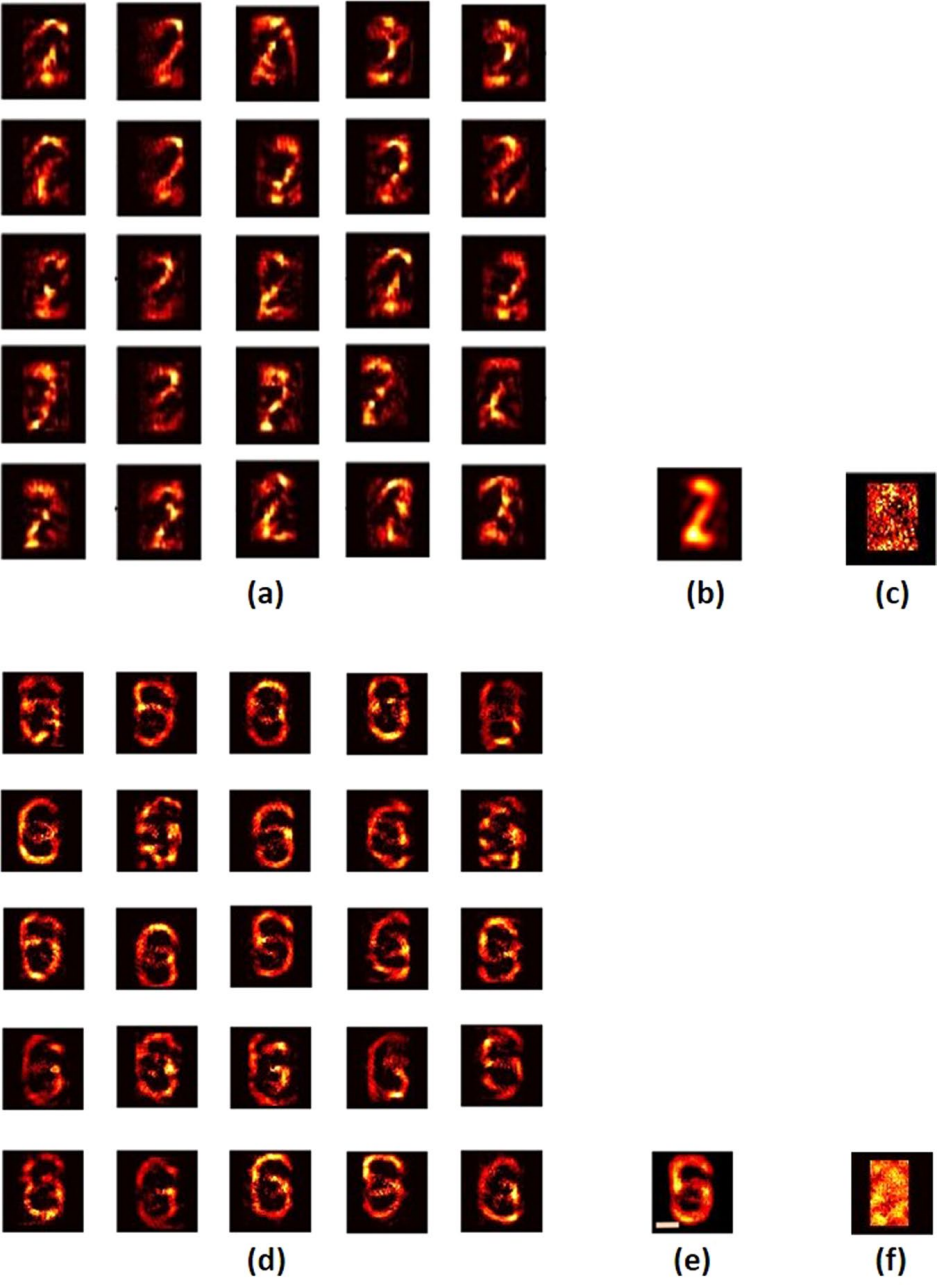
(**a,d**) Reconstruction results with bipolar intensity patterns using the phase retrieval method (**b**) and (**e**) Averaged reconstruction result. (**c**) Reconstruction result with the diffuser and only QPM on the SLM for the digit ‘2’ and (**f**) for the digit ‘6’.

The same phase retrieval algorithm was applied on the autocorrelation of the bipolar matrix. The reconstruction results for both the objects ‘2’ and ‘6’, with 25 different initial random are shown in Fig. 12(a,d), respectively. The results of Fig. 12(a,d) were obtained from the first 25 experiments without omitting any result. Furthermore, for both groups of images, averaging was done over the complex images to improve the quality of results. In order to average, the center of mass was calculated for every reconstructed image, and each image was shifted such that all the images had a common center of mass. The averaged reconstruction results of all the images are shown in Fig. 12(b,e). It should be emphasized that the entire digital process of running the phase retrieval algorithm 25 times, and the averaging procedure, were done on the same two camera shots. Thus, if the digital process is done off-line on a set of shot pairs, recording the scene can be done in real-time. To better appreciate the improvement in the quality of images provided by the system developed in this study, Fig. 12(c,f) show the reconstruction results for both the objects ‘2’ and ‘6’ with the same retrieval algorithm obtained without the CPM (but with the QPM).

## Summary and Conclusions

We have proposed and demonstrated a noninvasive technique to image through a scattering layer, using a two user-controlled phase coded masks with two camera shots. The explanation to the success of the two-shot method is as following; in each intensity pattern measured by the camera after passing through the scattering media there is a dominant bias function, which does not contain any information on the hidden object, but on the other hand, obscures the relatively small signal, which does contain the information about the object autocorrelation. The difference between the two measured intensity patterns created from two independent, but statistically similar CPMs, contains only negligible remains from the bias function, because there is not much difference between the two bias functions. After the elimination of the bias, the autocorrelation of the difference between the two measured intensities mostly contains the information of the covered object autocorrelation as is indicated by Eq. (10), and hence the object itself can be reconstructed by the phase retrieval algorithm operating on this autocorrelation.

The method can be easily applied for imaging through different types of scatterers by accurately engineering the autocorrelation profile of the PSF, using suitable QPM to determine the width of the autocorrelation peak and proper CPMs to determine the width of the background distribution. It is important to keep the width of the autocorrelation peak as small as possible and its sharpness as high as possible. As the peak is sharper, the input to the phase retrieval algorithm is more similar to the autocorrelation of the observed object, and hence, the final outcome for the algorithm is more similar to the object. The width of the background distribution is not significant in our method because the background is removed in any case. However, for single shot methods^24^ in which the background is removed digitally under the assumption that its distribution is constant, the width of the background can be calibrated such that its distribution can be constant, at least over the area of the observed objects.

The method is different than the method proposed in^24^ by several aspects. First, in the proposed method there are two camera shots captured through two different independent CPMs. That makes the overall two scattering media to be in a weak correlation between them (in comparison to their autocorrelation peaks). Two shots, in comparison to a single shot, add more information about the hidden object to the algorithm and have the effect of averaging and consequently the effect of noise reduction. Moreover, unlike^24^, our method of background removal from the autocorrelation is not limited to a constant background only. Any background function can be removed as long as the statistics of CPMs and of the scattering layers remains the same between the two camera shots. The technique of background removal in our case is done only by subtracting the two camera patterns obtained from the two independent CPMs. Therefore, there is no need to crop the autocorrelation with any kind of digital window in a trial and error procedure before the phase-retrieval algorithm^24^.

Another difference from the lensless system of^24^ is that the proposed system is equipped with a diffracted lens displayed on the SLM in arrangement that makes the PSF equal to the magnitude square of the Fourier transform of the scatterer product. Unlike^24^, this arrangement guarantees that the sharpness of the PSF autocorrelation peak is dependent on the diameter of the system aperture and independent on the scattering degree of the scatterers.

The proposed technique needs two exposures and apparently is slower than single-shot methods^24^. However, for SLM with refresh frequency standard of 60 Hz, an acquisition of 30 frames per second can be regarded as a real time imaging. Therefore, a video of dynamic hidden object can be recorded assuming the iterative phase retrieval process of the image reconstruction is performed off-line as many times as needed on the entire pairs of captured raw patterns. The averaging on the many outcomes from the phase retrieval algorithm is also done off-line without slowing the double-shots capturing process.

We believe that the proposed technique will be useful for imaging through scattering layers with lesser complexity and time compared to the existing techniques. In this study, the method is only applied for 2D imaging. Therefore, additional studies are required to improve the system performances toward 3D imaging systems.
